# Large community-acquired Legionnaires’ disease outbreak caused by *Legionella pneumophila* serogroup 1, Italy, July to August 2018

**DOI:** 10.2807/1560-7917.ES.2020.25.20.1900523

**Published:** 2020-05-21

**Authors:** Marino Faccini, Antonio Giampiero Russo, Maira Bonini, Sara Tunesi, Rossella Murtas, Monica Sandrini, Sabrina Senatore, Anna Lamberti, Giorgio Ciconali, Serafina Cammarata, Eros Barrese, Valentina Ceriotti, Sonia Vitaliti, Marina Foti, Gabriella Gentili, Elisabetta Graziano, Emerico Panciroli, Marco Bosio, Maria Gramegna, Danilo Cereda, Carlo Federico Perno, Ester Mazzola, Daniela Campisi, Gianuario Aulicino, Silvana Castaldi, Antonietta Girolamo, Maria Grazia Caporali, Maria Scaturro, Maria Cristina Rota, Maria Luisa Ricci

**Affiliations:** 1 Agency for Health Protection of Metropolitan Area of Milan (ATS), Milan, Italy; 2 These authors contributed equally to this article and share first authorship; 3 Direzione Generale Welfare, Unità Organizzativa Prevenzione, Lombardy Region, Milan, Italy; 4 Department of Laboratory Medicine, Hospital Niguarda, Milan, Italy; 5 Department of Biomedical Sciences for Health, Post Graduate School in Public Health, University of Milan, Milan, Italy; 6 Fondazione IRCCS Ca’ Granda Ospedale Maggiore Policlinico, Milan, Italy; 7 Department of Infectious Diseases, Istituto Superiore di Sanità, Rome, Italy

**Keywords:** *Legionella pneumophila*, legionnaires’ disease, outbreak, Italy

## Abstract

In July 2018, a large outbreak of Legionnaires’ disease (LD) caused by *Legionella pneumophila* serogroup 1 (Lp1) occurred in Bresso, Italy. Fifty-two cases were diagnosed, including five deaths. We performed an epidemiological investigation and prepared a map of the places cases visited during the incubation period. All sites identified as potential sources were investigated and sampled. Association between heavy rainfall and LD cases was evaluated in a case-crossover study. We also performed a case–control study and an aerosol dispersion investigation model. Lp1 was isolated from 22 of 598 analysed water samples; four clinical isolates were typed using monoclonal antibodies and sequence-based typing. Four Lp1 human strains were ST23, of which two were Philadelphia and two were France-Allentown subgroup. Lp1 ST23 France-Allentown was isolated only from a public fountain. In the case-crossover study, extreme precipitation 5–6 days before symptom onset was associated with increased LD risk. The aerosol dispersion model showed that the fountain matched the case distribution best. The case–control study demonstrated a significant eightfold increase in risk for cases residing near the public fountain. The three studies and the matching of clinical and environmental Lp1 strains identified the fountain as the source responsible for the epidemic.

## Background

*Legionella pneumophila* (Lp) is a Gram-negative bacterium responsible for a severe pneumonia named Legionnaires’ disease (LD). This infection represents 1.9% of all community‐acquired pneumonia cases, 4.0% of hospitalised cases and 7.9% of cases requiring admission to intensive care units [1]. The case fatality rate of LD ranges from 5% to 30% during outbreaks but can reach up to 50% in nosocomial cases or if antibiotic treatment is delayed [2]. The European Legionnaires’ disease Surveillance Network (ELDSNet) has reported an increase in age-standardised LD notification rates in the period 2011 to 2017 [3]. The same trend has been observed in Italy, with incidence rates increasing from 1.56 per 100,000 in 2011 to 4.9 per 100,000 in 2018 [4]. LD occurs predominantly in the elderly with chronic lung disease; immunosuppression and smoking as the most important risk factors. The incubation period ranges between 2 and 10 days from the, often nonspecific, initial symptoms. Infection occurs through inhalation of aerosols produced by contaminated water systems [3]. Outbreaks have been linked to a variety of aerosol‐producing devices, such as cooling towers, evaporative condensers and spa pools [5,6].

Improvements in diagnosis and surveillance may partly explain the increase in reported LD cases worldwide, however, several studies have suggested that higher atmospheric temperatures and changes in rainfall patterns may play a significant role [7-9]. Moreover, age-standardised rates are increasing, with a greater number of fragile individuals who are at higher risk of acquiring the infection [4].

Bresso is a town (3.38 km^2^ with 26,285 inhabitants) located near Milan in Lombardy, the region with the third highest LD incidence in Italy (10 cases per 100,000 people in 2018) [10]. In 2014, an LD cluster occurred in Bresso involving six cases within a period of 20 days. All cases were men aged 58–78 years, one of whom died. The only clinical isolate available was typed as ST23. The source of infection was not identified.

In July 2018, a new and larger outbreak of LD occurred in Bresso, involving 52 cases. The aim of this paper was to report epidemiological, microbiological and environmental investigations and describe factors that contributed to the outbreak.

## Outbreak detection

In Bresso, the number of LD cases reported in the period 2015 to 2017 was between one and three cases per year. Thus, when the Agency for Health Protection of the Metropolitan Area of Milan (ATS) received the notification of three LD cases occurring in citizens living in Bresso between 16 and 17 July 2018, the suspicion of an epidemic cluster arose immediately. Additional cases were soon notified and the suspicion was confirmed. A multidisciplinary team including epidemiologists, public health operators, medical doctors and microbiologists was established to control the outbreak and to conduct epidemiological and environmental investigations.

## Methods

### Case definition and epidemiological investigation

A probable outbreak-associated case was defined as a person with confirmed or probable LD according to the European Union (EU) case definition [11] with symptom onset between 10 and 31 July 2018, who lived in, or visited, the outbreak area (the town of Bresso) in the 10 days before symptom onset. Confirmed nosocomial cases or cases who had travelled outside Bresso for the entire incubation period were not considered.

An epidemiological investigation was performed and information regarding demographic, clinical and risk factors was collected. A map of the routes and places visited in the city of Bresso during the incubation period was drawn for each case. The information collected was used for geolocalisation studies and routes and places were plotted using ArcGIS software (esri, Redlands, United States (US)).

### Environmental investigation

All sites identified as potential sources of the disease were inspected and sampled. The sites were chosen based on: the place of residence and main locations frequented by the cases (e.g. malls, recreational facilities and squares) and highly crowded outdoor areas with aerosol-producing devices (e.g. cooling systems, sprinklers and fountains). The municipal water main line was also sampled at selected points. All potential sources were entered into the geographical information system (GIS).

### Microbiological analysis of clinical and environmental samples

LD cases were diagnosed by both urinary antigen test (Binax Now; Alere-Abbott, Scarborough, US) and culture of respiratory secretions or pulmonary tissues. For culture examination, we used agar plates with buffered charcoal yeast extract (BCYE-α; Thermo Fisher Diagnostics Limited - Altrincham, United Kingdom) and BCYE with selective antibiotic (MWY; Thermo Fisher Diagnostics).

Water samples were tested by both real-time PCR and culture. Real-time PCR assays were performed using a validated commercial kit (iQ-Check Screen Legionella spp and iQ-Check Screen L. pneumophila; Biorad, Marnes-la-Coquette, France), according to ISO/TS 12869, 2012 [12]. Water samples positive for Lp by qualitative real-time PCR (automatic cycle threshold (CT), positive ≤ 43 CT) were also analysed by culture in order to isolate *Legionella* strains. For culture, 1 L of water was analysed according to ISO 11731:2017 using a detection limit of 100 colony-forming units (CFU)/L) [13]. In some cases, to increase *Legionella* recovery, biofilms or sediments were sampled and analysed according to ISO 11731:2017.

Colonies from clinical and environmental samples were identified by latex agglutination test (Thermo Fisher Diagnostics Limited - Altrincham). All Lp serogroup 1 (Lp1) colonies identified were typed using monoclonal antibodies (MAb) of the Dresden Panel [14] and genotyped by sequence-based typing (SBT) [15]. 

### Case-crossover design

To evaluate the associations between heavy rainfall and LD cases, a unidirectional case-crossover design [16,17] was adopted. For each subject, the case-day was identified as the day of LD symptom onset. Control-days were defined as the period ranging from 10 to 3 days before the case-day. For example, for a subject with symptoms starting on 15 July, the case-day was identified as 15 July and the control days ranged from 5 to 11 July 2018.

Each case-day was matched with its control-days so that adjustment for usual confounding, such as age and sex, was assured by design. The occurrence of extreme precipitation events (defined as days with a precipitation higher than 31 mL/h) was compared between case-days and control-days. Thirty mL of daily cumulative rainfall was used as a cut-off point to identify heavy rainfall, and a dummy variable was used as exposure. Climatological and air quality components were included as covariates.

Hourly environmental data concerning precipitation, air temperature and relative humidity, measured between July and August 2018, were obtained from a monitoring station of the Regional Environmental Protection Agency (ARPA), located 2 km from Bresso. Mean daily data on particulate matter 10 µm or less in diameter (PM10) and on nitrogen dioxide (NO_2_) were obtained from a monitoring station located in Bresso [11].

### Case–control study

A retrospective case–control study was also performed. Cases were defined as reported in the case definition and up to four controls were selected for each case [18,19]. Controls were randomly selected from the local residency register and matched with cases for sex, age and history of chronic pathology (renal failure, diabetes, etc). Each control was matched with a case for all the conditions included as matching variables, and residence was geolocalised using the ArcGIS software. To investigate the potential source of infection, we analysed place of residence and main meeting points such as malls, fountains and shops. Exposure was defined as the distance of each case and its controls from each potential source positive for Lp1, hypothesising that the prevalence of cases living close to the source was different from the prevalence of controls. Distance from each source was divided in tertiles and the third tertile was used as the reference group.

### Aerosol dispersion investigation

Dispersion models were used to estimate the exposed population following a potential release and to infer potential source sites from the pattern of observed infections. We investigated the transport and dispersion of aerosol during the infection period (5–20 July 2018) using the LAPMOD modelling system [20]. This is a three-dimensional, non-stationary, Lagrangian particle model used to simulate the atmospheric dispersion of inert or radioactive gases and aerosols, over complex terrain, emitted from the potential source of infection.

To improve the resolution of terrain topography and land use, the meteorological input of the LAPMOD model was the output field generated by the diagnostic meteorological model CALMET [21] from 1 to 31 July. The geophysical variables necessary for the LAPMOD model estimation of deposition flows (i.e. roughness or soil occupation category) also come from CALMET.

CALMET was generated using wind temperature, direction and speed values at various altitudes of the troposphere, provided by the radiosonde at Milan Linate airport, the prognostic meteorological field produced by the weather research and forecasting model and the surface temperature from Bresso ARPA monitoring station.

The weather field used was validated by reconstructing the wind rose at the nearest ARPA control unit, not included in the calibration phase.

Inhalation of *Legionella*-contaminated droplets occurs when the aerosol size is smaller than 10 μm [5,22]. For this reason, we investigated the dispersion of PM10 from a plausible pipe diameter. Emission height, output velocity and temperature were introduced in the model according to source characteristics. We simulated two different types of dispersion: cooling towers and fountains. For cooling towers, we hypothesised a pipe diameter of 1 m positioned on the roof of the corresponding building [23]. Output velocity was 3 m/s, emission rate was 100 g/s and, being hot emissions, a temperature 10 °C higher than the temperature recorded by the nearest ARPA control units, assessed every hour. For fountains, we simulated a cold emission of 100 g/s positioned at ground level and a diameter of 1 m. Since the real emission rates were not available, we hypothesised an emission rate of 100 g/s for each potential source [23]. Dry and wet depositions were simulated in both models. We estimated the mean concentration of emissions and defined levels of intensity from very low to very high. Plumes were visualised using ArcGIS.

### Statistical analysis

Odds ratios were calculated using conditional logistic regression with LD diagnosis as outcome, distance from potential source in case–control and extreme precipitation in case-crossover studies.

Climatological and air quality components such as NO_2_, PM10, temperature and relative humidity, were considered as potential confounders.

All statistical analyses were performed using SAS version 9.4.

### Ethical statement

Ethical approval was not necessary because the data used for the study were collected as part of the infectious disease surveillance programmes defined by national legislation.

## Results

### Epidemiological investigation

**Figure 1:**
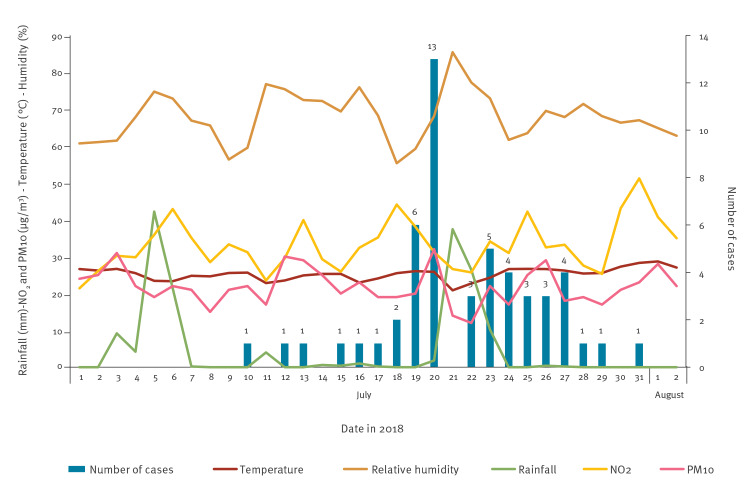
Confirmed cases of Legionnaires’ disease by date of symptom onset, Bresso, Italy, 10–31 July 2018 (n = 52)

Fifty-two confirmed LD cases among residents in Bresso were notified between 10 and 31 July, with a peak on 20 July (Figure 1).

Temperature, relative humidity, rain measure and pollution (PM10 and NO_2_) are also shown.

The mean patient age was 73 years (range: 33–95; median: 75) and 32 (61.5%) were men. Forty-six cases presented at least one individual risk factor for LD; 41 patients had an underlying disease and 22 patients had two or more chronic conditions. Eighteen cases reported cigarette smoking, which was the only risk factor in six cases. Forty cases were hospitalised and five died. The case fatality rate was 9.6%.

Most of the cases reported to have visited the central area of the town during the incubation period, which also coincided with the place of residence of many cases (n = 41).

### Microbiological investigation

All 52 LD cases were confirmed by urinary antigen test and Lp1 was isolated by culture of respiratory secretions in four cases. The SBT and MAb typing was carried out both for clinical and environmental strains. Of the four Lp1 ST23 human strains two were Philadelphia and two were France-Allentown subgroup. Typing data of Lp environmental isolates are reported in the Table.

**Table:** *Legionella pneumophila* serogroup 1 colonies isolated from different environmental sites, Bresso, Italy, 10–31 July 2018 (n = 8 isolates)

Site	Sampling point	MAb subgroup	ST
Public fountain (Site A)	Collection basin	France/Allentown	23
House 1	Shower	Oxford	1
House 2	Shower	Benidorm	2,695
Hotel (Site C)	Cooling tower pond	Philadelphia	1
Industry 1 (Site B)	Cooling tower pond	Bellingham	37
Industry 1 (site B)	Cooling tower pond	Bellingham	1
Industry 1 (site B)	Cooling tower pond	Olda	1
Industry 2	Tap of toilet	Philadelphia	1

MAb: monoclonal antibody; ST: sequence type.

Overall, 101 sites (52 patients’ homes and 49 potential sources) were sampled and 598 water samples were collected. 

Five of 52 patients’ houses were found to be contaminated by Lp and only two houses resulted culture-positive for Lp1 (3,100 and 26,000 CFU/L). Real-time PCR was positive in four residences, including the two positive by culture.

Seven of 49 sites identified as potential sources were found positive for Lp by culture, while real-time PCR gave positive results in 10 sites including the seven positive by culture. Two of 11 inspected cooling towers resulted positive for Lp1 with 1,000 CFU/L and 6,500 CFU/L. Moreover, a decorative fountain located in a public garden of the town, where many inhabitants stopped or passed by, was sampled on 25 July 2018 and was found positive for Lp1 (1,000–2,000 CFU/L). Sampling of the fountain was repeated 1 month and 3 months later and was found contaminated with Lp1 at 2,000 CFU/L and 50,000 CFU/L, respectively. Three additional decorative fountains located in the outbreak area were sampled: two were negative (< 100 CFU/L) and one positive for Lp5. The municipal water system was sampled at 10 different points, identified as critical, and these samples were all negative (< 100 CFU/L).

### Case-crossover design

A total of 52 LD case-days and 364 control-days were included in the analysis. Precipitation values ranged from 0.0 to 42.2 mL per day, with an average value of 3.7 mL per day over the study period and a cumulative value of 82.6 mL. Two high precipitation events (> 31 mL/h [24]) occurred during the outbreak period: 5 to 21 July 2018. Daily temperatures ranged between 20.9 °C and 26.6 °C with an average of 24.5 °C. The average relative humidity was 69.8% (range: 56.3–85.4). The daily PM10 ranged from 12 μg/m^3^ to 32 μg/m^3^, with an average of 21 μg/m^3^. The daily NO_2_ ranged from 25.7 μg/m^3^ to 39.9 μg/m^3^, with an average of 32.3 μg/m^3^. Adjusted analysis revealed that extreme precipitations, occurring 5 and 6 days before symptom onset, were associated with a fourfold increase in LD risk (OR = 4.03; 95% confidence interval (CI): 0.76–21.3 and OR = 3.58; 95% CI: 0.80–16.1, respectively).

### Case–control study

For each of the 48 cases, four matched controls were identified, i.e. 48 cases and 192 controls were included in the analysis. Of 101 sites analysed, Lp1 was only isolated from four non-residential sources: The mean distance of the public fountain from the place of residence was 391 m for cases and 646 m for controls (OR for first vs third tertile: 8.69; 95% CI: 3.04–24.83); the mean distance to Industry 1 was 777 m for cases and 661 m for controls (OR for first vs third tertile: 2.95; 95% CI: 1.15–7.59); the mean distance to the hotel was 1,859 m for cases and 2,153 m for controls (OR for first vs third tertile: 11.43; 95% CI: 3.46–37.69); and the mean distance to Industry 2 was 688 m for cases and 540 m for controls (OR for first vs third tertile: 0.90; 95% CI: 0.32–2.58).

### Aerosol dispersion investigation

**Figure 2:**
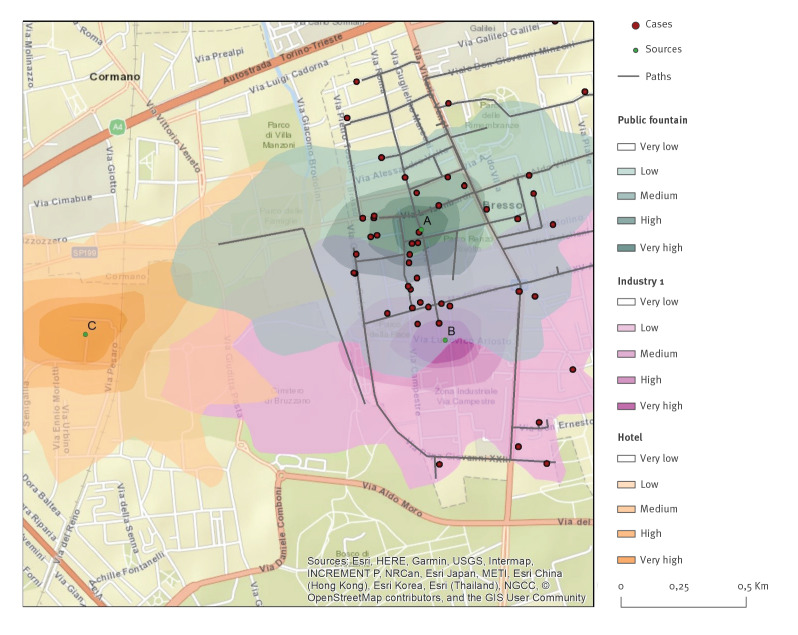
Mapping of Legionnaires’ disease cases: paths collected through individual questionnaires and aerosol dispersion model with potential sources’ plumes, Bresso, Italy, 10–31 July 2018 (n =52)

Given that Industry 2 was not associated with LD in our case–control study, we limited the aerosol dispersion investigation to the public fountain (Site A), Industry 1 (Site B) and the hotel (Site C). A different map of aerosol dispersion over the area of interest was generated for each site contaminated with Lp1. The modelled dispersions were used to establish the proportion of cases exposed to the various potential sources by either living close to or visiting a location during the incubation period. The plume modelled from the public fountain (Site A) showed the best fit with the distribution of the cases (Figure 2).

Public fountain (site A), cooling tower of Industry 1 (site B) and hotel (site C). Different colours represent the intensity of concentration within each plume.

## Outbreak control measures

At the beginning of the investigation, emergency control measures were requested for all sites resulting positive by real-time PCR. In particular, the public fountain was immediately deactivated on 19 July, while cleaning and disinfection of the cooling towers were requested on 9 August. Once culture results were also available, disinfection was only requested for culture-positive sites. Owners of the sampled residences and sites at risk were informed of the sampling results and were asked to follow the best practices for *Legionella* control. No more cases occurred demonstrating the efficacy of control measures applied. 

## Discussion

The outbreak described here was the largest caused by Lp1 in Italy to date. A thorough environmental investigation excluded the municipal water system as source of the outbreak, since *Legionella* was undetectable in all samples collected. Nevertheless, the percentage of positive samples collected from cases’ places of residence was small and no clinical strains were available for patients whose houses’ samples resulted positive. Thus, matching from clinical and environmental isolates could not be performed.

The most common reported sources of *Legionella* outbreaks are cooling towers [25,26]. In Italy, two LD outbreaks caused by cooling towers occurred in 1995 and 2003, and another occurred in the period 2005 to 2008, for which hidden cooling towers were suspected [27,28]. In the outbreak in Bresso, the SBT analysis showed that the human strains matched only with the environmental isolates from a small public fountain that had the same sequence type (ST23).

LD outbreaks have seldom been associated with decorative fountains [29,30]; in 2007, O'Loughlin et al. showed that a small fountain, without obvious aerosol-generating capability, was identified as the source of 18 LD cases, and that removal of the fountain halted disease transmission. In addition to sociodemographic factors and associated chronic conditions, many factors influence LD transmission rates: bacterial concentration, distance from the source, high humidity, low atmospheric pressure and extreme rainfall events [7,8,23,31].

The Bresso fountain was characterised by water recirculation, with little or no apparent aerosol production. Therefore, it was difficult to explain how the cases could have been infected. To identify the source of the outbreak, we carried out three studies.

The case-crossover study suggested that the heavy rainfalls which occurred 5 to 6 days before the onset of disease were associated with a fourfold increase in LD risk, even if not statistically significant. This time lag was slightly shorter than those reported in previous studies: (1 week [31] and 6–10 days [32]) and may have been related to particular atmospheric conditions in this area, such as the combination of temperature and high humidity that may promote *Legionella* spread.

The case–control study, performed to evaluate the association between the cases’ places of residence and the distance from identified contaminated sites, showed a significant eightfold increase in risk for cases living near the public fountain. The OR for the hotel was 11.43; however, the strains isolated from the hotel cooling tower had a different ST than the clinical isolates. In addition, the hotel was located further the cases’ places of residence than the fountain, and no cases were detected among hotel staff or guests.

Finally, an analytical study of the aerosol dispersion model applied to the potential sources showed that the fountain was the most probable source of infection.

A literature review has shown that the distance of *Legionella* transmission in an outbreak situation can vary from 500 m to 12 km [33]. In the Bresso outbreak, the potential source was identified at an average distance of 600 m from the cases.

A Lagrangian approach was used to model the discrete phase transport of bio-aerosol deposition. We hypothesised that the interaction between the high bacterial load in the recirculating water system that fed the fountain basin and the extreme rainfall of 5 and 21 July generated a bio-aerosol of contaminated particles which dispersed from the fountain, as simulated by the model.

We therefore consider it plausible that *Legionella* bio-aerosols emitted from the fountain could have entered the surrounding buildings. This conclusion is reinforced by the fact that outdoor bio-aerosols have high penetration efficiency [5] and more than half the outbreak cases were sedentary individuals older than 75 years who reported spending most of their time at home or in indoor gathering places. The potential dispersion area of the public fountain was densely inhabited by cases.

All the studies conducted pointed to the public fountain as a potential source of the outbreak. In fact, the cleaning, disinfection and deactivation of the fountain on 19 July, halted the outbreak.

It is difficult to explain why, after several years of operation, the fountain suddenly caused a large outbreak, but several elements must be considered. Firstly, the fountain is located in a public garden with connections to several unused water pipes. Secondly, the summer of 2018 was characterised by very high temperatures (hourly maximum temperature: 35 °C) and humidity (hourly maximum humidity: 86%) throughout the months of June and July [34] and by two extreme rainfall events. This combination could have created fertile ground for *Legionella* growth and persistence.

As reported in a recent review, warm weather, rain and higher relative humidity may have an impact on the survival of airborne *Legionella*, as it has been shown to survive better at a relative humidity of 65% and less so at 30% or 90% relative humidity. Our meteorological data showed that relative humidity ranged from 60 to 80% throughout the outbreak period, increasing the risk of acquiring LD [35].

In conclusion, although at the beginning of the investigation, the public fountain did not appear as a plausible source of infection, the findings of all three studies, combined with the matching of the fountain isolates with the clinical isolates, identified the fountain as responsible for the outbreak.

### Study limitations

The main limitation of the study is that only few clinical isolates were available, so that we could not ascertain if sources contaminated with other ST also contributed to the outbreak.

Another limitation was the information input to calculate the aerosol dispersion map. Changes in exit velocity and model characteristics resulted in large variations in emission concentrations and dispersion area. Furthermore, we investigated only the cases’ places of residence and not all places visited, therefore underestimating exposure for some cases. A critical aspect was also the lack of robust methodology to ensure consistent bio-aerosol dispersion models.

Lastly, in the case-crossover study, although OR were not statistically significant, they were very close to 1 and the upper CI limits were very high. Thus, increasing the sample size would probably have yielded significant results.
